# Induction of Corneal Endothelial-like Cells from Mesenchymal Stem Cells of the Umbilical Cord

**DOI:** 10.3390/ijms232315408

**Published:** 2022-12-06

**Authors:** Eun Ah Ye, Ho Seok Chung, Yoonkyung Park, Jeong Hye Sunwoo, Whanseo Lee, Jin Kim, Hungwon Tchah, Hun Lee, Jae Yong Kim

**Affiliations:** 1 Department of Ophthalmology, Asan Medical Center, University of Ulsan College of Medicine, Seoul 05505, Republic of Korea; 2 Department of Ophthalmology, Dankook University Hospital, Dankook University College of Medicine, Cheonan 31116, Republic of Korea

**Keywords:** umbilical cord, mesenchymal stem cells, corneal endothelial cells, rabbit model of corneal endothelial dysfunction

## Abstract

Because of the limited differentiation capacity of human corneal endothelial cells (CECs), stem cells have emerged as a potential remedy for corneal endothelial dysfunction (CED). This study aimed to demonstrate the differentiation of human umbilical cord-derived mesenchymal stem cells (UC-MSCs) into CECs and to investigate the efficacy of MSC-induced CEC injection into the anterior chamber in a rabbit model of CED. Human UC-MSCs were differentiated into CECs using medium containing glycogen synthase kinase 3β inhibitor and two types of Rho-associated protein kinase inhibitors. In the MSC-induced CECs, CEC-specific proteins were identified through immunohistochemistry and changes in CEC-specific gene expressions over time were confirmed through quantitative RT-PCR. When MSC-induced CECs were injected into a rabbit model of CED, corneal opacity and neovascularization were improved compared with the non-transplanted control or MSC injection group. We also confirmed that MSC-induced CECs were well engrafted as evidenced by human mitochondrial DNA in the central cornea of an animal model. Therefore, we demonstrated the differentiation of UC-MSCs into CECs in vitro and demonstrated the clinical efficacy of MSC-induced CEC injection, providing in vivo evidence that MSC-induced CECs have potential as a treatment option for CED.

## 1. Introduction

In the field of ophthalmology, permanent blindness caused by corneal endothelial dysfunction (CED), is a must-solve problem. During the developmental process, human corneal endothelial cells (CECs) differentiate from mesenchymal stem cells via cranial neural crest cells and form a monolayer with a thickness of approximately 4 μM [1]. CECs, unlike other cells comprising the cornea, have a limited ability to differentiate, resulting in irreversible damage in surgical injuries or congenital diseases such as Fuchs’ dystrophy. 

Corneal endothelial transplantation, such as Descemet’s stripping endothelial keratoplasty or Descemet’s membrane endothelial keratoplasty, is commonly used to treat corneal endothelial abnormalities that do not involve the corneal epithelium or stroma. However, the number of donors is limited, and re-transplantation is occasionally required because of graft rejection or endothelial cell loss during long-term observation. Therefore, corneal endothelial transplantation is insufficient as a perfect solution for patients with CED. As a result, stem cells have emerged as a potential remedy to these limitations. Treatment via injection of the endothelial-like cells induced from embryonic stem cells or induced pluripotent stem cells (iPSCs), directly into the anterior chamber has been attempted, with encouraging results [2,3,4,5,6].

Mesenchymal stem cells (MSC) are multipotent stem cells that can differentiate into multiple types of cells such as osteoblasts, chondrocytes, myocytes, and adipocytes [7,8]. It has been reported that MSCs have immunological privilege because of their immunomodulatory properties. [9,10]. Moreover, there are no concerns with regard to teratogenic potentials of iPSCs. MSCs obtained from umbilical cord (UC) tissues are the youngest and most primitive cells with the fastest growth rate. Thus, they have been frequently used in research for clinical applications. Several studies have demonstrated that human umbilical cord-derived mesenchymal stem cells (UC-MSCs) can differentiate into corneal epithelial cells or corneal stromal cells (to some extent), although the differentiation into CECs is comparatively insufficient [11,12,13,14].

This study aimed to demonstrate the differentiation of UC-MSCs into CECs and to investigate the efficacy of injection of MSC-induced CECs into the anterior chamber in a rabbit model of CED. 

## 2. Results

### 2.1. In vitro Differentiation of MSCs into Corneal Endothelial Cells

**Figure 1:**
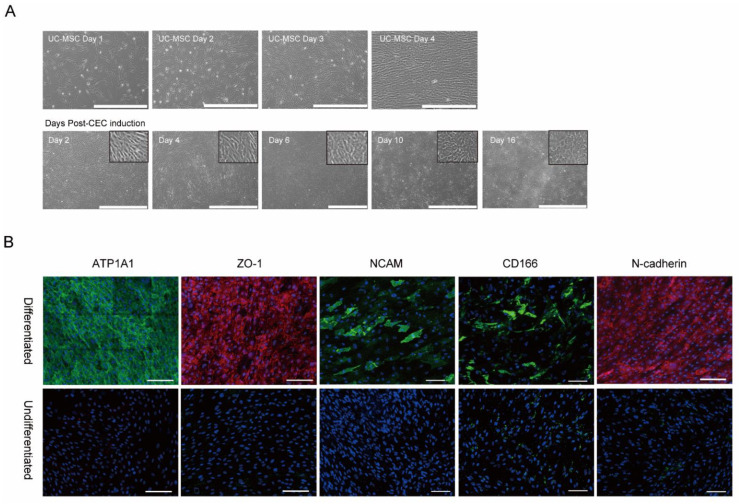
Generation of corneal endothelial cells from umbilical cord-derived mesenchymal stem cells. (**A**) Phase-contrast microscopic images taken from days 1 to 4 of UC-MSC culture and taken 2, 4, 6, 10, and 16 days after CEC induction. Scale bars = 1 mm (**upper**) or 500 µm (**lower**). (**B**) Immunocytochemistry of pump function protein Na^+^/K^+^ ATPase α1 (ATP1A1), zona occludens-1 (ZO-1; a tight-junction protein), NCAM (CD56), CD166, and N-cadherin as characteristic markers of CECs at 16 days after CEC induction. Cell nuclei were counterstained with DAPI. Scale bars = 100 μm.

Phase-contrast microscopy during CEC differentiation showed likely CECs on day 6 and CECs exhibiting CEC-like hexagonal/polygonal morphology on day 10 (Figure 1A). We investigated the expression of the CEC-specific proteins, ATP1A1, ZO-1, NCAM, CD166, and N-cadherin in MSC-induced CECs through immunocytochemical staining. Most of the cells strongly expressed ATP1A1, ZO-1, and N-cadherin and relatively smaller populations expressed NCAM and CD166 (Figure 1B).

**Figure 2:**
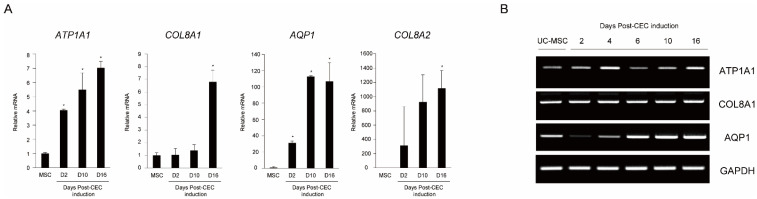
Quantitative RT-PCR and RT-PCR. (**A**) Quantitative RT-PCR (qRT-PCR) analysis of the expression of representative MSC, and CEC genes. (**B**) Gel image of RT-PCR showing MSC and CEC-related gene expression. (* *p* < 0.05).

To determine whether differentiation to CEC was successful, we collected cells from MSC and MSC-induced CECs on differentiation days 2, 10, and 16, and measured the expression of CEC markers (*ATP1A1*, *COL8A1*, *AQP1*, and *COL8A2*) by qRT-PCR. Gene expression of *ATP1A1* and *AQP1* significantly increased 2 days after CEC differentiation and then increased thereafter, while that of *COL8A1* and *COL8A2* significantly increased 16 days after CEC differentiation (Figure 2A). The PCR results of *AQP1* showed a similar pattern of gene expression with qRT-PCR (Figure 2B).

**Figure 3:**
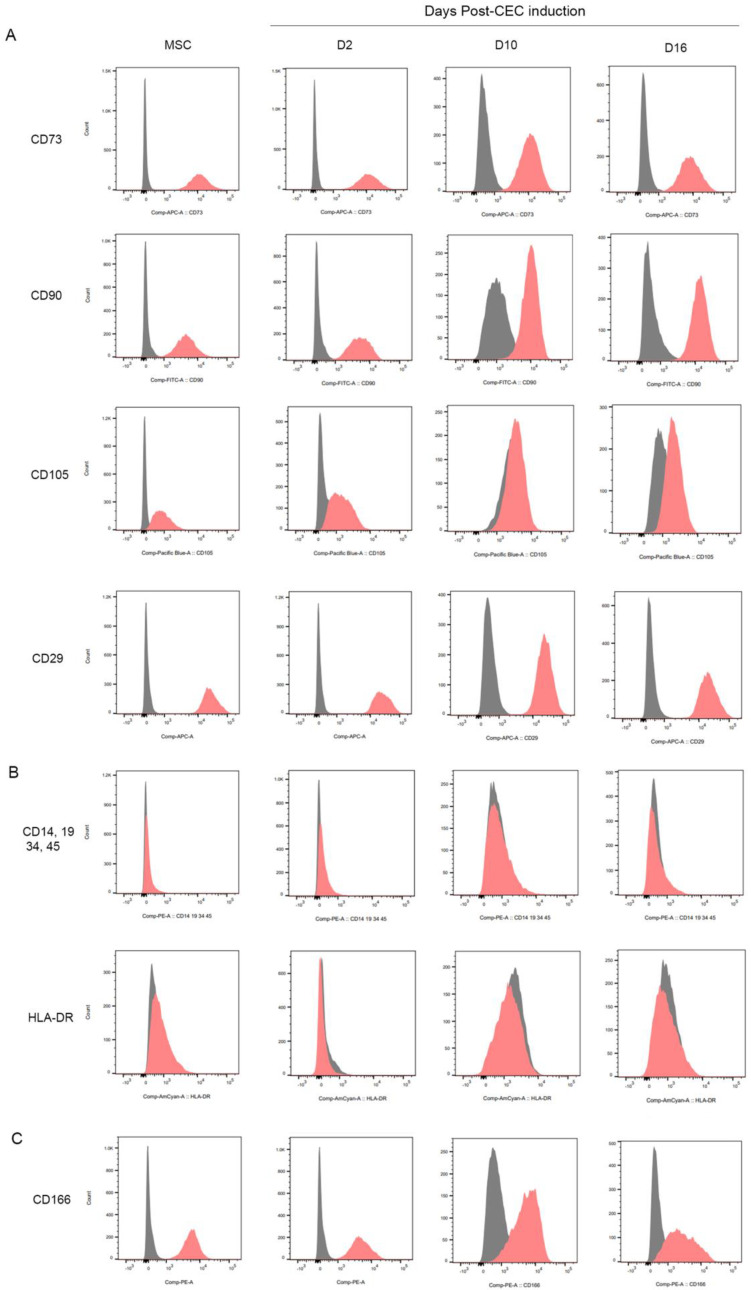
FACS results for MSC-associated surface markers and human CEC-associated markers. (**A**) Results for positively expressed surface markers in human umbilical cord MSCs. (**B**) Results for negatively expressed surface markers in human umbilical cord MSCs. (**C**) Results for marker used to identify CECs.

FACS data showed that the human-derived UC-MSCs had positive surface expression of MSC-associated surface markers such as CD73, CD90, CD105, and CD29 (Figure 3A) and negative CD14, CD19, CD34, CD45, and HLA-DR expression (Figure 3B). UC-MSC-induced CECs had positive surface expression of MSC-associated surface markers CD73, CD90, CD29 and negative CD14, CD19, CD34, CD45, and HLA-DR expression (Figure 3A,B). The expression level of CD105 has been changed as the CEC induction has processed through D16, suggesting the differentiation processes induced decreased levels of a MSC surface marker (CD105) expression. In addition, MSC-induced CECs were positive for CD166, which has been used to identify human CECs (Figure 3C).

### 2.2. In Vivo Injection of MSCs and MSC-Induced CECs into a Rabbit Anterior Chamber

**Figure 4:**
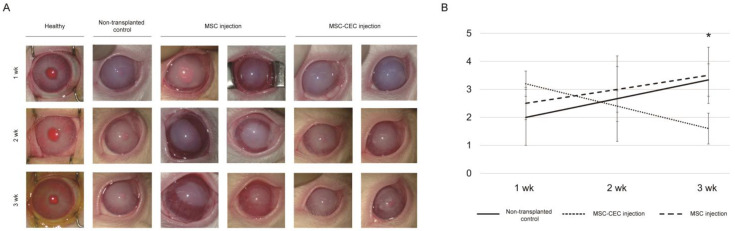
Slit-lamp microscope image of the healthy group, non-transplanted control group, MSC injection group, and MSC-induced CEC injection group at 1, 2, and 3 weeks after cell transplantation. (**A**) The MSC-induced CEC injection group exhibited a lower corneal opacity score than the non-transplanted control group at 3 weeks, showing less neovascularization than the MSC injection group. (**B**) Corneal opacity score over time. The MSC-induced CEC injection group had the lowest corneal opacity score at 3 weeks after cell transplantation. (* *p* < 0.05).

Based on results from differentiation studies, we transplanted MSC-induced CECs into a rabbit model of CED. The animals were divided into a group transplanted with MSC or MSC-induced CECs (cell transplantation group) and a non-transplantation control group and all observed for 3 weeks. At 1, 2, and 3 weeks after injection, corneal neovascularization and corneal opacity worsened over time in the non-transplanted control and the MSC injection groups. We found that the MSC-induced CEC injection group showed more severe corneal opacity than the non-transplanted control group at 1 week after injection; however, it was improved at 3 weeks after injection (Figure 4B). When assessing corneal opacity on a scale of 0–4 before and after cell transplantation, there was a statistically significant improvement in corneal opacity. Compared with the non-transplanted control group, transplantation of MSC-induced CECs reversed cloudy corneas into relatively clear corneas, which was evident at 3 weeks after injection (Figure 4A). The corneal opacity score of the MSC-induced CEC injection group was significantly lower than that of the non-transplantation control group (1.60 ± 0.55 to 3.33 ± 0.58, *p* = 0.020) and MSC injection group (1.50 ± 0.71 to 3.50 ± 1.00, *p* = 0.026) (Table 1); the MSC-induced CEC injection group also showed less neovascularization than the MSC injection group.

### 2.3. Engraft of Human Origin MSC-Induced Corneal Endothelial Cells

**Figure 5:**
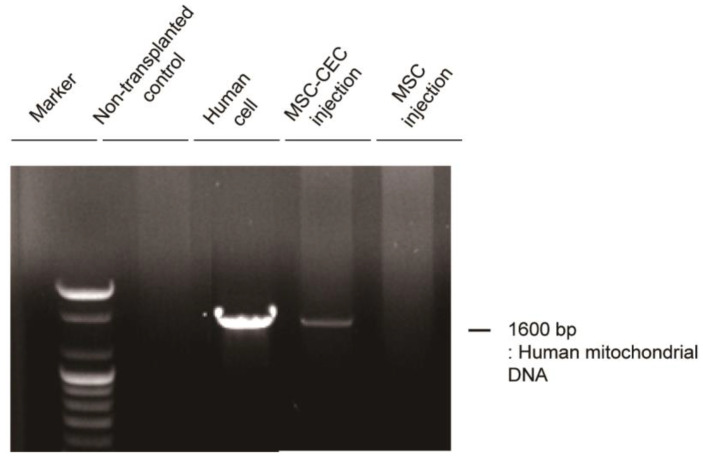
Engraft of human origin MSC-induced CECs after transplantation of MSC-induced CECs into corneal endothelial disease models. Gel image of RT-PCR showing human mitochondrial DNA to confirm the presence of human cells in corneal tissue after transplantation. Since human mitochondrial DNA was detected in the corneal tissue of the transplantation group, MSC-induced CECs were engrafted in the cornea after transplantation.

To determine whether MSC-induced CECs were transplanted into the cornea region, we performed a human mitochondrial DNA PCR reaction using DNA extracted from the cornea 3 weeks after transplantation to confirm that the cells were derived from transplanted human MSC-induced CECs as opposed to rabbit cells (Figure 5). Human mitochondrial DNA was found in the cornea of the rabbits transplanted with MSC-induced CECs, whereas there were no specific bands found in human cells in the non-transplanted control group and MSC injection group.

## 3. Discussion

In this study, we successfully differentiated UC-MSCs into corneal endothelial-like cells, as evidenced by the results from immunocytochemistry confirming the expression of CEC-specific proteins after UC-MSC induction. We used qRT-PCR to confirm whether CEC-specific gene expression markers increased over time. Corneal opacity and neovascularization were improved when MSC-induced CECs were injected, compared to the non-transplanted control or MSC injection group in an in vivo experiment. In addition, MSC-induced CECs were well engrafted in the rabbit cornea after cell transplantation, as demonstrated by the presence of human mitochondrial DNA in the central cornea of a CED animal model following injection of MSC-induced CECs. 

CED can develop after eventful or uneventful cataract surgery, or because of various corneal endothelial dystrophy. CED is one of the common causes of corneal endothelial transplantation in developed countries, and it is a group of diseases with significant socioeconomic implications [15]. Corneal endothelial cells are arrested in the G1 phase in vivo by several mechanisms and maintained in a non-replicative state [16]. As a result of CEC injury, peripheral normal endothelial cells migrate to the central cornea, resulting in a decrease in endothelial cell density [17]. A previous study demonstrated clinical improvement when CECs extracted from a donor cornea were directly expanded in vitro and injected into CED patients [18,19]. However, this approach of direct expansion of CECs has limitations in that CECs may have poorer proliferation capacity in vitro, and contamination by keratocytes with higher proliferative capacity can be problematic [20]. For that reason, corneal endothelial cell differentiation from non-ocular-derived stem cells, such as MSCs, embryonic stem cells, and induced pluripotent stem cells, has been explored, with several recent achievements reported [6,20,21].

MSCs play a role in the normal development of CECs and have intrinsic paracrine effects, such as anti-inflammatory and anti-fibrotic properties [22]. Because of these properties, studies on the effect of MSCs on the treatment of corneal epithelial insufficiency have been conducted. In a rabbit model of alkali-induced corneal injury, MSC-derived factors have been demonstrated to reduce corneal inflammation and neovascularization and to promote epithelial wound healing [23]. Because of its more primitive features, UC-derived MSCs are emerging as a treatment strategy for a variety of systemic diseases. Moreover, the presence of UC-MSCs has been observed to promote the repair and regeneration of the corneal epithelial cells [24,25]. There was strong evidence that corneal stromal cell markers were expressed following adequate differentiation from the MSCs into corneal cells, as well as partial evidence for differentiation into corneal epithelial cells [21]. However, it was mentioned that the evidence for differentiation into CECs was deemed insufficient since only preliminary data were reported [26,27]. One previous in vitro study found that UC-MSCs can “home” into areas of injured CECs and can themselves be altered into CEC-like cells, which could be an advantage of employing MSCs in cell therapy of CED [26]. However, in our study, human mitochondrial DNA was not found in the MSC injection group. Moreover, corneal improvement after MSC injection in the rabbit model of CED was insufficient when compared to the MSC-CEC injection group. It is possible that the microenvironment in rabbits’ anterior chambers was not conducive to the migration of MSCs to the injured CECs or differentiation of MSCs into CECs. 

In the present study, the differentiation of UC-MSCs into CECs was demonstrated by increased expression of the relative mRNA levels of *ATP1A1, COL8A1, AQP1*, and *COL8A2* over time after CEC differentiation. These genes serve as CEC-specific markers, ATP1A1 and AQP1 are the major components of CEC pump action, and COL8A1 and COL8A2 are ECM components of CEC. In addition, protein expression of CEC markers was shown using a panel of antibodies against ATP1A1, ZO-1, NCAM, CD166, and N-cadherin. From the flow cytometry analysis, there was a slight decrease in the levels of CD166 between D10 and D16. We cannot exclude the possibility that CD166 displayed a differential expression pattern on the MSCs during CEC-induction. Our immunocytochemistry results in Figure 1B also showed that relatively smaller populations of cells were immunoreactive for CD166 at day 16 of CEC induction as compared to the expression of other CEC markers. As a MSC positive marker, CD105 also showed a differential pattern of expression as time progressed during CEC induction, while the levels of other MSC positive markers have not changed noticeably.

Yamashita et al. recently reported that corneal endothelial-like cell differentiation was induced using a medium containing glycogen synthase kinase (GSK) 3-β inhibitor, and that after transplantation of these cells into rabbit eyes with bullous keratopathy, corneal thickness and transparency recovered [28]. Several studies have shown that GSK 3-β inhibitor can promote endothelial differentiation from mouse cornea-derived precursors, human skin-derived precursors, and eye field stem cells by activating the Wnt pathway [29,30,31,32]. Using the same method as previous research, we assumed that it was possible to induce and culture corneal endothelial-like cells with pump action [28].

Rho-associated protein kinase (ROCK) inhibitor can improve corneal adhesion and cell proliferation by increasing the transition from G1 to S phase by activation of the PI 3-kinase/Akt signaling, and thus have been supplemented in previous studies about corneal cell therapy and demonstrated effectiveness [33,34,35,36]. In the CED animal model, 10 mM Y-27632 eye drops promoted corneal endothelial wound healing and increased the expression of N-Cadherin and Na^+^/K^+^ ATPase compared with the control group [37]. In a human pilot study conducted in the same group, Y-27632 reduced corneal edema and increased endothelial cell density [37]. Meanwhile, another ROCK inhibitor, H-1152, improved CEC migration and proliferation more than Y-27632 in vitro and improved corneal endothelial wound healing in vivo after topical application of 2.5 μM H-1152 [38]. We attempted to maximize the CEC proliferation in this study by using both types of ROCK inhibitors, Y-27632 and H-1152. 

In addition, we used polyvinyl alcohol (PVA) instead of fetal bovine serum (FBS) which can cause an acute immune response after transplantation because of xeno-contamination. FBS can contaminate cells during cell culture and differentiation, resulting in the presence of xenoantigens and infectious agents that may provoke graft versus host disease [39,40]. The addition of serum introduces a complex protein mixture of unknown composition and exhibits wide variability between batches [41]. Furthermore, the use of FBS in cell culture and differentiation has been associated with safety concerns due to the risk of increased intraocular pressure and cell proliferation through systemic dissemination [20]. There may also be differences between batches [40]. Based upon the in vitro and in vivo results, PVA can replace FBS and improve the differentiation of MSCs into CECs.

Regarding the cell therapy of CED, a variety of injection methods have been developed. In the reports of Yamashita et al., the recipient cornea was punched, and a CEC sheet was attached to the donor cornea and transplanted in the same method as penetrating keratoplasty [28]. In the study by Kinoshita et al., endothelial cells from donated human corneas were cultured and injected with supplementation of a ROCK inhibitor to increase CEC density and improve visual acuity in patients with bullous keratopathy [19]. Although this approach has limited attachment and allows cells to escape through the trabecular meshwork, it is a more straightforward method than sheet transplantation and is relatively adequate for clinical use. A method of injecting immunomagnetic umbilical cord blood endothelial progenitor cells was also introduced; however, more research is needed because teratogenicity has been described as a result of the use of magnetic nanoparticles in animal models [42]. In the present study, Kinoshita’s approach of injecting CEC suspension into the anterior chamber was used. Corneal opacity decreased over time in the MSC-induced CEC injection group compared with that in the non-transplanted control group, and human mitochondrial DNA was found in the central cornea of our rabbit animal model after MSC-induced CEC transplantation. Moreover, no evidence of transplantation-related adverse effects was seen in any of the rabbits. Our findings, therefore, provided in vivo evidence that in the rabbit CED animal model, UC-MSCs can be used as progenitor cells for corneal endothelial cell allografts. These findings came from an in vivo xenograft method in which cells of human origin were transplanted into rabbits. Hence, when interpreting the results, it should be cautious about generalizing these findings to other species, especially humans. This limitation can be compensated by comparing the results with new in vitro experimental techniques (3D reactors, organ-on-a-chip, or 3D printers).

## 4. Methods and Materials

### 4.1. Corneal Endothelial Cell Differentiation

Human UC-MSCs were provided by the Asan Stem Cell Center (Asian Institute for Life Sciences, Seoul, Republic of Korea). The UC-MSCs were cultured in Dulbecco’s modified Eagle’s medium/nutrient mixture of F12 (Life Technology, Grand Island, NY, USA), with 10% FBS (Life Technology), and antibiotics (100 U/mL penicillin, 100 µg/mL (0.01%) streptomycin, 0.025 µg/mL (0.0000025%) amphotericin B). The medium was changed every 2 days. MSCs were directly visualized by phase-contrast microscopy and photographs were taken up to the fourth day after harvest. From the fourth day of culture, the induction of CECs from MSCs was conducted by modifying a previously published procedure [28]. Initial culture was performed at a density of 3 × 10^5^ cells per 75-cm^2^ flasks, and UC-MSCs reached >95% confluence in 4–5 days on FNC (FNC coating mix; United States Biological, Salem, MA, USA)-coated culture plates. For induction to CECs, the culture medium was replaced to CEC-induction medium, and MSCs were incubated for subsequent 16 days. The composition of the culture medium for inducing CEC differentiation was as follows: human endothelial-SFM (Life Technology, Grand Island, NY, USA) supplemented with 0.1% polyvinyl alcohol (PVA, Sigma-Aldrich, St. Louis, MO, USA), 0.5 mM BIO (glycogen synthase kinase 3β inhibitor, Alexis Corporation, Lausen, Switzerland), 10 µM Y-27632 (Sigma-Aldrich), 1% insulin-transferrin-selenium (Life Technologies), 0.02 mg/mL 2-phosphate ascorbic acid (Stemcell Technology, Cambridge, MA, USA), 1 μM SB431542 (Selleckchem, Houston, TX, USA), 2.5 μM H-1152 (Tocris, Abington, UK), and 0.2 mg/mL CaCl_2_ (Sigma-Aldrich). The medium was changed every 3 to 4 days. MSC-induced CECs were directly visualized by phase-contrast microscopy, and photographs were taken on days 2, 4, 6, 10, and 16 following the initiation of induction.

### 4.2. Immunocytochemical Staining of MSC-Induced Corneal Endothelial Cells

MSC-induced CECs were washed three times with PBS, fixed in 10% neutral buffered formalin overnight in a refrigerator (2–8 °C), and then blocked with 0.1% bovine serum albumin (Sigma-Aldrich) and 5% donkey serum (Jackson ImmunoResearch Laboratories Inc., West Grove, PA, USA) at room temperature for 30 min. MSC-induced CECs were then incubated with primary antibodies for zonular occludens 1 (ZO-1; 1:100, 61-7300; Life technologies), alpha 1 sodium-potassium ATPase (ATP1A1; 1:500, ab76020; Abcam, Inc., Cambridge, MA, USA), CD166 (1:200, 559260; BD Biosciences, San Jose, CA, USA), NCAM-1/CD56 (1:200, MAB24081; R and D systems, Minneapolis, MN, USA), and N-cadherin (1:150, ab5581; Abcam, Inc.) overnight at 4 °C. Cells were washed three times with PBS and incubated with rhodamine-conjugated donkey anti-rabbit IgG and/or FITC-conjugated donkey anti-mouse IgG (1:100; Jackson ImmunoResearch Laboratories Inc.). Cells were again washed three times with PBS for 10 min each and stained with 4’-6-diamidino-2-phenylindole (DAPI) (Vector Laboratories, Inc., Burlingame, CA, USA) for 1 min to counterstain cell nuclei. The fluorescence signals were detected with either a laser-scanning confocal microscope (Olympus, Tokyo, Japan) or a fluorescence microscope (EVOS, Life Technologies).

### 4.3. RT-PCR and Quantitative RT-PCR (qRT-PCR) 

Total RNA from MSC-induced CECs was extracted using TRIzol reagent (Invitrogen Carlsbad, CA, USA). Complementary DNA was synthesized using a kit (Superscript III; Invitrogen). qRT-PCR was performed with Power SYBR Green PCR Master Mix (Applied Biosystems, CA, USA) on a Step One ABI Real-Time PCR System (Applied Biosystems). CEC-associated markers, such as *ATP1A1*, *COL8A1*, *AQP1*, and *COL8A2* at 2, 4, 6, 10, and 16 days post-CEC induction were measured. The mRNA levels were normalized to GAPDH. Primer sequences are listed in Supplementary Table S1.

### 4.4. Flow Cytometry Analysis

The MSC-induced CECs from passage 2 or 3 were harvested, washed twice with PBS, incubated for 10 min with 2 mL TrypLE (Invitrogen) at 37 °C, and collected. Cells were then stained for MSC or CEC markers using the Miltenyi Human MSC Phenotyping Kit (130-125-285; Miltenyi Biotec Ltd., Auburn, CA, USA) and antibodies against CD29 and CD166. 5 µL of each antibody cocktail was added at room temperature for 30 min. The cells were then washed twice by adding 3 mL flow cytometry buffer and centrifuging at 300× *g* for 10 min. The cells were then run through a Becton Dickinson FACSCanto I flow cytometer and the flow cytometry plots were analyzed using FACSDiva software. Antibodies used to detect CD29 and CD166 were as follows: PE Mouse Anti-Human CD166 (559263), APC Mouse Anti-Human CD29 (559883), PE mouse IgG1, K Isotype control (555749), APC mouse IgG1, K Isotype control (555751; BD Biosciences, San Jose, CA, USA).

### 4.5. In Vivo Cell Transplantation Experiments

Experiments on live vertebrates were conducted in strict accordance with the relevant national and international guidelines regarding animal handling as mandated by the Institutional Animal Care and Use Committee (IACUC) of the University of Ulsan College of Medicine (Seoul, Republic of Korea). The committee reviewed and approved the animal study protocol (2019-12-184).

New Zealand white rabbits (*n* = 12), each weighing 1.8 to 2.2 kg, were placed in standard rabbit cages and housed under good environmental control. The room temperature was maintained at 24 °C with a 12-h light/dark cycle. After 7 days of acclimation, the animals underwent the procedure, performed by an experienced physician. All interventions were performed under anesthesia, and all efforts were made to minimize discomfort. All rabbits were anesthetized with an intramuscular injection of 5 mg/kg Zoletil (Virbac Korea, Seoul, Republic of Korea) and 2 mg/kg Rompun (Bayer Korea, Seoul, Republic of Korea). Then, topical anesthesia was performed with 0.5% proparacaine hydrochloride (Alcaine^®^; Alcon Laboratories, Inc., Fort Worth, TX, USA). The CED model was developed as follows: after mydriasis of the rabbit’s left eye, the anterior chamber was filled with viscoelastic (Healon; Johnson and Johnson Vision, Santa Ana, CA, USA) through a paracentesis incision, and the Descemet’s membrane was scored and stripped in a circular pattern under the area of the epithelial marking (6 mm) using a reverse Sinskey hook. After irrigation of the anterior chamber using balanced salt solution (Alcon Laboratories, Inc.), 4 mg gentamicin (Shin Poog Pharmaceutical Co., Seoul, Republic of Korea) and 0.5 mg dexamethasone (Jeil Pharmaceutical Co., Seoul, Republic of Korea) were injected into the subconjunctival space. 

We injected MSC-induced CECs (*n* = 4, 1 × 10^6^ MSC-CECs in 150 μL of PBS supplemented with 100 μM of Y-27632) into the anterior chamber through a paracentesis incision in the MSC-induced CEC injection group. Meanwhile, we injected MSCs (*n* = 4, 1 × 10^6^ MSCs in 150 μL of PBS supplemented with 100 μM of Y-27632) into the anterior chamber through a paracentesis incision in the MSC injection group. The rabbits in the cell transplantation group were positioned with the left eye down and kept for 3 h under respiratory anesthesia to allow the injected cells to adhere to the corneal posterior surface. The rabbits were evaluated for up to 3 weeks. In the non-transplantation group (control; *n* = 4), rabbits were evaluated for up to 3 weeks after Descemet’s membrane stripping only. We assessed corneal opacity on a scale of 0–4 (0 = none; 1 = mild turbidity, iris texture evident; 2 = moderate turbidity, iris texture unclear; 3 = severe turbidity, pupil faint; and 4 = severe turbidity, pupil not visible) using a slit-lamp microscope, before and 1, 2, and 3 weeks after cell transplantation. Rabbit corneas were harvested 3 weeks after cell transplantation. All procedures conformed to the Association for Research in Vision and Ophthalmology (ARVO) Statement for the Use of Animals in Ophthalmic and Vision Research (ARVO Animal Policy).

### 4.6. Human Mitochondrial DNA Detection

Total DNA was isolated from enucleated eyes 3 weeks following cell transplantation using the PicoPure^TM^ DNA extraction Kit (Life Technologies). A PCR reaction was conducted using 2× PCRBIO HS Taq Mix Red (PCR Biosystems, London, UK) with 300 ng of DNA in a total volume of 20 μL. The reaction was performed as described in the kit instructions with annealing at 56 °C for 20 s.

### 4.7. Statistical Analysis

All experiments were performed on at least three independent biological samples and the data are presented as means ± standard deviations (SD). Statistical analysis was performed with GraphPad Prism 6.0 software (GraphPad Software, CA, USA). Comparisons of three or more data sets were done using a one-way or two-way ANOVA followed by Bonferroni’s multiple comparison correction. Two-group comparisons were made using two-tailed Student’s *t*-tests. *p* < 0.05 was considered statistically significant.

## 5. Conclusions

In conclusion, UC-MSCs were shown to be able to differentiate into corneal endothelial-like cells in vitro, and when the MSC-induced CECs were injected in vivo into a rabbit model of CED, together with a ROCK inhibitor, corneal opacity and neovascularization were improved compared with the MSC injection group or non-transplanted control group.

## Figures and Tables

**Table 1:** Mean corneal opacity score for each group at weeks 1, 2, and 3.

	Non-Transplanted Control (*n* = 4)	MSC Injection (*n* = 4)	MSC-Induced CEC Injection (*n* = 4)
Week 1	2.0 ± 1.0	2.5 ± 0.6	3.2 ± 0.4
Week 2	2.7 ± 1.5	3.0 ± 0.8	2.4 ± 0.5
Week 3	3.3 ± 0.6	3.5 ± 1.0	1.6 ± 0.5

## Data Availability

The dataset used during the current study are available from the corresponding author on reasonable request.

## Supplementary Materials

The supporting information can be downloaded at https://www.mdpi.com/article/10.3390/ijms232315408/s1.
