# Supplementary material for: Induction of Corneal Endothelial-like Cells from Mesenchymal Stem Cells of the Umbilical Cord
Source: Int J Mol Sci. 2022 Dec 6;23(23):15408. doi: 10.3390/ijms232315408 (PMC9739507; doi:10.3390/ijms232315408)
Supplement: Supplementary file 1 [file ijms-23-15408-s001.zip › ijms-1801657-supplementary.pdf]

Table S1. Primer sequences used for PCR.

Gene	Primer sequence
AQP1	F: 5′-ATGGCCAGCGAGTTCAAGAAGAAG-3′
	R: 5′-GGTTCTGCCCTGGGCTTCAAAT-3′
COL8A1	F: 5′-CCT GGG TCA GCA AGT ACC TC-3′
	R: 5′-TTG TTC CCC TCG TAA ACT GG-3′
COL8A2	F: 5′-CTG GTT TGG ATG GGC TTC CT-3′
	R: 5′-CTC ATC GAA GGC CCC AGG AG-3′
ATP1A1	F: 5′-ACA GAC TTG AGC CGG GGA TTA-3′
	R: 5′-TCC ATT CAG GAG TAG TGG GAC-3′
GAPDH	F: 5′-GTATCGTGGAAGGACTCATGACCA-3′
	R: 5′-TAGAGGCAGGGATGATGTTCTGGA-3′
Human mitochondrial DNA	mt8709F: 5′-CAA CAC TAA AGG ACG AAC CTG A-3′
	mt10171R: 5′-TCG TAA GGG GTG GAT TTT TC-3′
